# Determination of Gonyautoxin-4 in Echinoderms and Gastropod Matrices by Conversion to Neosaxitoxin Using 2-Mercaptoethanol and Post-Column Oxidation Liquid Chromatography with Fluorescence Detection

**DOI:** 10.3390/toxins8010011

**Published:** 2015-12-30

**Authors:** Marisa Silva, Verónica Rey, Ana Botana, Vitor Vasconcelos, Luis Botana

**Affiliations:** 1 Department of Biology, Faculty of Sciences, University of Porto, Rua do Campo Alegre, Porto 4619-007, Portugal; marisasilva17@gmail.com; 2 Interdisciplinary Center of Marine and Environmental Research–CIMAR/CIIMAR, University of Porto, Rua dos Bragas, 289, Porto 4050-123, Portugal; 3 Department of Analytical Chemistry, Science Faculty, University of Santiago of Compostela, Lugo 27002, Spain; veronica.rey@rai.usc.es (V.R.); anamaria.botana@usc.es (A.B.); 4 Department of Pharmacology, Veterinary Faculty, University of Santiago de Compostela, Lugo 27002, Spain; luis.botana@usc.es

**Keywords:** paralytic shellfish poisoning, toxins, post-column oxidation fluorescence, interfering matrix peaks, thiol compounds

## Abstract

Paralytic Shellfish Toxin blooms are common worldwide, which makes their monitoring crucial in the prevention of poisoning incidents. These toxins can be monitored by a variety of techniques, including mouse bioassay, receptor binding assay, and liquid chromatography with either mass spectrometric or pre- or post-column fluorescence detection. The post-column oxidation liquid chromatography with fluorescence detection method, used routinely in our laboratory, has been shown to be a reliable method for monitoring paralytic shellfish toxins in mussel, scallop, oyster and clam species. However, due to its high sensitivity to naturally fluorescent matrix interferences, when working with unconventional matrices, there may be problems in identifying toxins because of naturally fluorescent interferences that co-elute with the toxin peaks. This can lead to erroneous identification. In this study, in order to overcome this challenge in echinoderm and gastropod matrices, we optimized the conversion of Gonyautoxins 1 and 4 to Neosaxitoxin with 2-mercaptoethanol. We present a new and less time-consuming method with a good recovery (82.2%, RSD 1.1%, *n* = 3), requiring only a single reaction step.

## 1. Introduction

**Figure 1:**
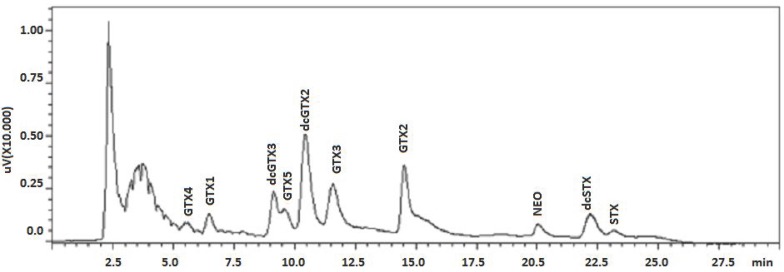
Paralytic Shellfish Toxins (PSTs) elution pattern in mussel matrix spiked with standards.

Paralytic Shellfish Toxins (PSTs) are neurotoxic alkaloids responsible for the Paralytic Shellfish Poisoning syndrome (PSP), produced mainly by three dinoflagellates genera: *Alexandrium*, *Pyrodinium* and *Gymnodinium*. This biotoxin group is composed of Saxitoxin (STX) and its analogues. To date, there are more than 57 known STX analogues, with the three main subgroups being: STX group (saxitoxin (STX), neosaxitoxin (NEO), decarbamoyl saxitoxin (dcSTX)), GTXs group (gonyautoxins 1–5 (GTX1, GTX2, GTX3, GTX4 and GTX5), decarbamoyl gonyautoxins 2–3 (dcGTX2, dcGTX3)) and C group (*N*-sulfocarbamoyl-gonyautoxins 1–4 (C3, C1, C2 and C4)) [1,2]. PSTs were first reported in the 1920s in the USA but now are reported worldwide, affecting the ecosystem, causing economic losses and human poisoning morbidity and mortality [3,4]. PSTs are fast acting toxins affecting the skeletal muscle by binding specifically to voltage gated sodium channels, inhibiting cell communication and causing paralysis [1,5]. The most common route of intoxication is through the ingestion of contaminated seafood, but thanks to monitoring programs and legislation, poisoning events have been scarce in the past 20 years [3,4]. While these measures are focused on bivalve mollusks, PSTs have been reported in other vectors, from crustaceans to gastropods and echinoderms; thus an updated risk assessment is currently required [6]. For many years the mouse bioassay (MBA) has been the standard reference method for the detection and quantitation monitoring [7]. Currently, due to ethical and technical issues associated with the MBA [8], chemical methods are preferred and mandated in regulatory monitoring in most countries, (e.g., EU legislation [7,9]). These methods depend on commercially available standard reference materials, which are: STX, NEO, GTX1,4, GTX2,3, GTX5, dcSTX, dcGTX2,3, C1, C2 and dcNEO. In 2006, a pre-column oxidation high performance liquid chromatography (HPLC) with fluorescence detection (FLD) method (Lawrence method) was integrated in the European directives as a reference method [9]. In 2011, a post-column oxidation high performance liquid chromatography (HPLC) with fluorescence detection (FLD) method (PCOX method) was validated by the Association of Official Analytical Chemists (AOAC International) [10]. The PCOX method presents several advantages regarding routine analysis in comparison to the Lawrence method [11]. However, high sensitivity to naturally fluorescent matrix interferences can pose some challenges [12]. The PCOX method is characterized by dividing the toxins into two groups: C group (C1, C2, C3 and C4) and GTXs and STXs group (GTX1, GTX2, GTX3, GTX4, GTX5, dcGTX2, dcGTX3, NEO, dcSTX and STX). In order to visualize the C toxins group, the standards are diluted in milliQ water, whereas for the GTXs and STXs group, the standards are diluted in PSP free shellfish tissue [13] and the elution pattern of the toxins starts with GTX4 and ends with STX (Figure 1).

In this study, several matrices were used: bivalves, gastropods, echinoderms, crustaceans and fish. However, as it was seen in previous studies with some scallop and oyster matrices [12,14], there can be naturally fluorescent interferences that co-elute with GTX4.This was found in this investigation with echinoderms and gastropods matrices. In the study of Baker *et al*. (2003) [15], five fluorescent compounds were identified with the same retention as GTX4 using HPLC-FLD, produced by two-strains of bacteria isolated from two *Alexandrium* species [15]. Rey *et al*. (2015) found that in both scallops and oysters, there are matrix interferences that hinder detection of GTX4. In addition, this work proved that the matrix interference varies with the date of harvesting and location and can be species specific [12]. In addition, some of the GTXs have been shown to be transformed reductively into STX or NEO by biologically available thiol reagents such as glutathione (GSH) [16]. This activity was also found in another thiol compound 2-mercaptoethanol (2-ME) [17], although the bioconversion of GTX1 and GTX4 into NEO in shellfish seems to happen specifically with GSH, as several researchers have observed [16,18]. These thiols selectively reduce O-sulfate group at C11 of GTX1 and GTX4 to form NEO; these compounds also convert GTX2,3 into STX [17].

To overcome this issue of naturally fluorescent matrix interferences, mixtures of GTX1, 4 were converted into NEO using 2-mercaptoethanol in samples which were suspected to have naturally fluorescent interferences.

## 2. Results and Discussion

Sakamoto *et al.* [17] converted GTXs to STXs with thiol compounds: GTX1 and GTX4 (4 nmol) were mixed with 1 mL of 8 mM 2-ME in 0.1 M phosphate buffer, pH 7.4. This mixture was then incubated at 70 °C for 2 h. The procedure used by Sakamoto *et al.* were performed in our laboratory but while GTX1 and GTX4 disappeared we detected no NEO formation as a result of the reaction. However, the theoretical yield of NEO should be 0.6 nmol (15% of conversion) after this reductive reaction, as it has been described [16] and these values are below the PCOX detection limit [12]. The low conversion rate may be due to the GTX and 2-ME conjugates. A second step is needed to complete the reaction, which consists of an incubation with 1M 2-ME at 100 °C for 10 min. so that conjugates of GTX1, GTX4 and 2-ME are converted into NEO. The percentage of recovery obtained was lower than expected, only 40% of NEO, whereas approximately 90% was expected, according to Sakamoto *et al.* [17]. In order to ensure the effectiveness of the transformation reaction, there must be an excess of thiol. Therefore, for the second step of the reaction, different concentrations of 2-ME in 0.1 M buffer phosphate were assayed: 1 M, 3 M and 5 M. No differences were observed with these variations in 2-ME concentration.

After taking the results into account, a different way of optimizing reaction conditions was attempted, whereby the incubation temperature and time were varied in a one-step reaction according to the following: 4 nmol of the standard mixture GTX1, 4 (3 nmol GTX1: 1 nmol GTX4) were mixed with 1 M 2-ME in 0.1 M phosphate buffer, pH 7.4. The mixture was heated in a water bath at 100 °C and different incubation times were tested: 10, 15, 20, 25, 30, 35 and 40 min (Table 1).

**Table 1:** Percentage of recovery of neosaxitoxin (NEO) after reaction of gonyautoxin (GTX)1, 4 with 2-mercaptoethanol (2-ME) 1 M in phosphate buffer 0.1 M, pH 7.4, and in gastropod matrix (average *n* = 3), and RSD % (Relative Standard Deviation).

Time (min)	100 °C Water Bath		100 °C Incubator		Gastropod Matrix	
	Average %	RSD %	Average %	RSD %	Average %	RSD %
10	59.5	0.8	64.5	2.8	62.2	1.2
15	55.3	0.3	58.4	1.7	69.7	1.5
20	62.6	1.0	58.9	1.0	71.9	1.1
25	81.1	1.2	61.4	1.0	80.2	1.5
30	82.2	1.1	50.5	1.4	84.3	1.3
35	65.8	1.8	47.7	1.0	71.1	1.4
40	64.8	1.3	31.0	2.4	68.0	1.8

The same procedure was carried out in an incubator at 100 °C and the times tested remained the same: 10, 15, 20, 25, 30, 35 and 40 min. In most cases, the results were lower than those obtained with the water bath (Table 1) and although the recovery percentages are a bit higher for 10 and 15 min, an optimal difference in recovery was observed at 30 min in the water bath. In general, the results show that the reaction is more effective with water bath compared to oven heating, and a higher percentage of transformed NEO was obtained after 30 min (Table 1).

**Figure 2:**
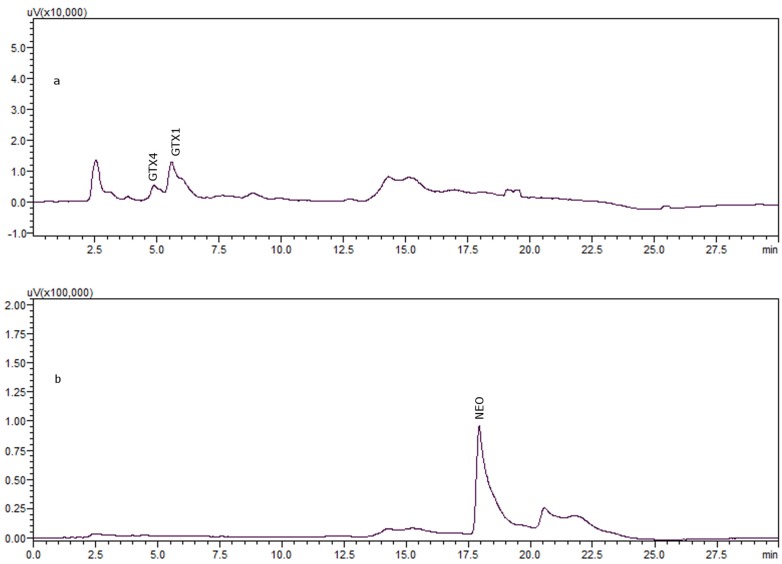
4 nmol of GTX1, 4 incubated with 1 M 2-ME in 0.1 M phosphate buffer, pH 7.4 during 30 min in water bath: (**a**) before incubation; (**b**) after incubation.

The reductive reaction was replicated three times (*n* = 3), in three different aliquots of the standard mixture and the average conversion percentage was 82.2% with Relative Standard Deviation (RSD) 1.1% with regard to repeatability. Although the results were not higher than those of previous studies [17], the reaction has been simplified from two steps to one, as shown in Figure 2a (before reductive reaction) and Figure 2b (after reductive reaction). The recovery was also tested in real matrix of gastropod: 4 nmol of the standard mixture GTX1, 4 (3 nmol GTX1: 1 nmol GTX4) were spiked into 100 μL of toxin-free matrix extract prepared according to what is described in Section 3.2.; 200 μL of 1 M 2-ME in 0.1 M phosphate buffer, pH 7.4 (see Equation (3)), were added and the recovery was tested at the times mentioned previously, in the water bath at 100 °C. Table 1 shows that the best recovery percentage is also for 30 min.

**Figure 3:**
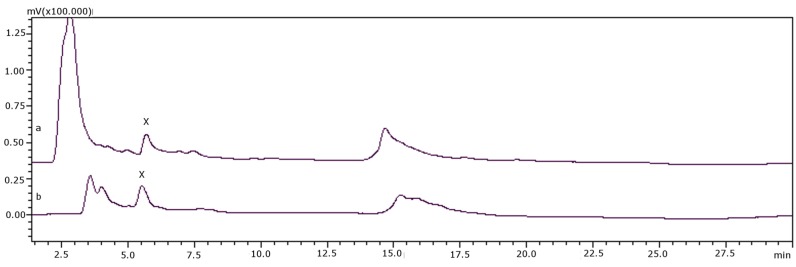
(**a**) Sample 477 (*S. haemostoma*) before transformation; (**b**) sample 477 (*S. haemostoma*) after transformation.

Samples suspected of containing GTX4 or a co-eluting naturally fluorescent interference were subjected to 2-ME reduction, as described in the sample preparation and transformation section. Table 2 shows tested samples and results. It was found that when the peak was GTX4, it disappeared and it was reduced to NEO. However, if the peak was caused by natural fluorescent components present in the sample, the size of the peak before and after the reductive reaction did not change, as described in the experimental section. Figure 3 shows the chromatograms of sample 477 (*S. haemostoma*) before incubation with 2-ME (Figure 3a) and after incubation with 2-ME (Figure 3b). This sample had a chromatographic peak named X, which could be either GTX4 or a natural fluorescent matrix interference that co-elutes with the toxin. After exposure to 2-ME, this peak was not reduced and there was no production of NEO, confirming that this peak was due to a naturally fluorescent matrix interference. Although there might be a co-elution of GTX4 and the matrix interference, no reduction in peak area was observed after reduction, so it can be deduced that only the interference is present.

**Table 2:** Tested samples and results before and after reaction with 2-ME.

Code	Type of Organism	Species	Origin/Date of Collection	Before Transformation (μM)		After Transformation (μM)	
				GTX4	GTX1	NEO	GTX4
351	Gastropod	*Umbraculum umbraculum*	Madeira Island/September 2012	1.7	1.1	1.3	0.2
353	Starfish	*Echinaster sepositus*	Madeira Island/September 2012	0.8	1.8	2.3	0.5
354	Gastropod	*Charonia lampas*	Madeira Island/September 2012	90.5	3.8	29.3	25.5
412	Starfish	*Ophidiaster ophidianus*	São Miguel Island, Azores/June 2013	17.3	3.7	4.4	0.7
424	Starfish	*O. ophidianus*	São Miguel Island, Azores/June 2013	17.0	-	4.6	4.6
428	Starfish	*Marthaterias glacialis*	São Miguel Island, Azores/June 2013	42.8	18.9	29.9	10.9
440	Starfish	*O. ophidianus*	São Miguel Island, Azores/June 2013	26.4	-	-	-
443	Gastropod	*Stramonita haemostoma*	Morocco/July 2013	428.6	-	-	-
454	Gastropod	*Cerithium vulgatum*	Morocco/July 2013	121.6	-	0.4	0.4
470	Gastropod	*Monodonta lineata*	Morocco/July 2013	3.9	-	2.5	2.5
474	Gastropod	*Onchidela celtica*	Morocco/July 2013	6.9	-	-	-
475	Gastropod	*C. lampas*	Morocco/July 2013	8.4	-	-	-
477	Gastropod	*Stramonita haemostoma*	Morocco/July 2013	2.5	-	-	-
483	Gastropod	*Gibbula umbilicalis*	Morocco/July 2013	13.3	-	-	-

**Figure 4:**
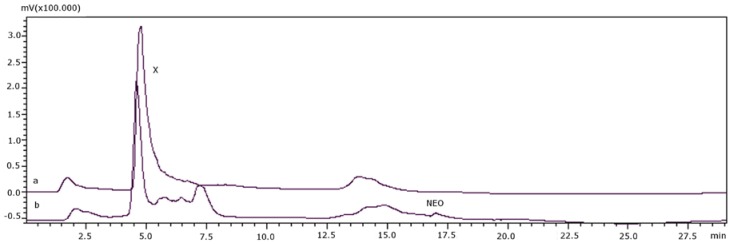
(**a**) Sample 454 (*Cerithium vulgatum*) before transformation; (**b**) sample 454 (*Cerithium vulgatum*) after transformation.

Figure 4 shows a positive sample, 454 (*Cerithium vulgatum*), before and after transformation (4a and 4b, respectively). The chromatographic peak for GTX4 (X) decreased but was not completely eliminated after the reaction. NEO was produced by the transformation, indicating that the peak was likely due to a combination of both GTX4 and a naturally fluorescent matrix interference.

In summary, when NEO was produced after the reductive reaction, the GTX4 peak was reduced. When NEO was not a product of the reduction, no change was observed in the GTX4 interference peak in the chromatogram, making peak assignment easier.

Table 2 summarizes all the samples tested with code, species and results before and after transformation.

After optimizing the conditions of the reductive reaction using 2-ME as a reductive agent, the same settings were tested with Glutathione (GSH). A solution of 0.2 M GSH in 0.1 M phosphate buffer, pH 7.4, was used to achieve transformation and it was found that after heating the mixture in a water bath for 30 min, GTX1,4 did not react with Glutathione.

While previous studies [16,17] demonstrated that both thiol reagents are able to transform GTXs in STXs, we found that, Glutathione was less effective than 2-Mercaptoethanol.

There is evidence that the action of intestinal bacteria mediates the transformation of GTXs analogs into STXs through GSH [18,19,20]. This conversion increases the toxic content of PSP-bearers, consequently increasing the toxicity in the food chain [6].

In the present study, the transformation reaction was used to confirm the presence and to quantify GTX4 in several samples.

The PCOX method used for monitoring PSTs requires the use of toxin-free shellfish extracts to prepare the calibration solution in order to overcome the matrix effect and also to facilitate data interpretation [13]. The post-column method poses some challenges regarding the co-elution of matrix peaks with toxin ones, hindering data analysis [12]; although it was optimized and validated for mussel, clam, scallop and oyster [13], and initially these four matrixes showed no naturally fluorescent matrix interferences, later studies [12,14] showed that both scallop and oyster have naturally fluorescent matrix interferences. GTX1 and GTX4 elute early off the chromatographic column [13], and this region has been shown to contain naturally fluorescent peaks in some matrices [12,14].

As toxin-free shellfish extracts of some of these matrices (echinoderm and gastropod) were not available, the performance of the method for these new matrices was not able to be assessed or validated. The conversion of GTX1 and GTX4 into NEO made it possible to detect and quantify GTX4 when it co-eluted with a naturally fluorescent matrix chromatographic peak. This is important because even though this method is only validated for bivalves, PSTs have been reported in other vectors [6] and could present a risk to the health of consumers.

It was observed that the transformation of GTX1 and GTX4 into NEO was most effective at a concentration of 1 M 2-ME in 0.1 M phosphate buffer (pH = 7.4), because when higher concentrations were tested (3 M and 5 M), there was no improvement in the yield of the reaction. While Sakamoto *et al.* found that it was necessary to have an excess of thiol compounds for the second step of the reaction [17], in this case there is an optimization of the protocol to a single one-step reaction using one solution of 2-ME. This is a remarkable improvement, although the conversion of GTX4 into NEO is not as complete as those shown in previous studies [17], which was 82.2% of the recovery found (*n* = 3, RSD = 1.1%).

The naturally fluorescent matrix peak was not affected by the addition of 2-ME allowing confirmation of non-detectable GTX4 when NEO was not produced, taking into account the decrease in the initial peak area when the toxin is present. This protocol represents a less time-consuming, easier and more economical way to overcome the challenge of co-eluting peaks in new shellfish matrices.

## 3. Experimental Section

### 3.1. Chemicals and Solutions

HPLC grade acetonitrile, ortho-phosphoric acid 85% (*v*/*v*), periodic acid, sodium hydroxide, nitric acid 65% (*v*/*v*), hydrochloric acid 37% (*v*/*v*), sodium dihydrogen phosphate (NaH_2_PO_4_) and disodium hydrogen phosphate (Na_2_HPO_4_) were acquired from Panreac Quimica S.A. (Barcelona, Spain). Heptane sulfonate, ammonium hydroxide 30% (*v*/*v*), trichloroacetic acid (TCA), 2-mercaptoethanol (2-ME) and glutathione (GSH) were purchased from Sigma Aldrich (Madrid, Spain).

Certified Reference Standard of NEO was purchased from CIFGA S.A. (Lugo, Spain), and GTX1,4 was provided by NRC (Institute for Marine Biosciences, Halifax, NS, Canada) for the identification of each toxin. A quality control standard of GTX1,4 from CIFGA S.A. was used for transformation reactions.

### 3.2. Sample Preparation and Transformation

Shellfish homogenate was extracted according to the procedure indicated in PCOX method [13]. The method involves a sample extraction with 0.1 M HCl and heating it in a boiling water bath for 5 min (adjust the pH previously if required between 2 and 4, preferably 3), an extract deproteination with 30% TCA and a neutralization with NaOH to bring the mixture pH between 2 and 4, preferably 3. Then the extract was filtered with 0.2 μm nylon membrane syringe filter (Whatman-Sigma Aldrich, Madrid, Spain) and injected into the system.

The samples containing the naturally fluorescent interferences were analyzed again after reductive transformation, as described below.

Mixtures of 4 nmol GTX1, 4 were mixed with 1 M 2-ME in 0.1 M phosphate buffer, pH 7.4. An aliquot of this mixture was analyzed by HPLC before reductive reaction to direct comparison with an aliquot after reductive reaction. These mixtures were heated at 100 °C for 30 min in water bath. An aliquot of the reduction reaction product was analyzed for toxin components by HPLC (Izasa Scientific, Madrid, Spain). Shellfish sample extracts underwent the same process and were analyzed by HPLC for toxin profile determination.

### 3.3. HPLC Toxins Identification

A modification of PCOX method was used [14] to identify PSTs. STX and GTXs groups were separated using a Zorbax Bonus-RP column (15 cm × 4.6 mm i.d., 3.5 μm particle size, part number 863668-901) from Agilent Technologies (Madrid, Spain), with column oven at 30 °C. Solvent A was composed by 8.25 mM heptane sulfonate and 5.5 mM H_3_PO_4_ aqueous solution adjusted to pH 7.1 using NH_4_OH 28%–30%. Solvent B composition was 8.25 mM heptane sulfonate, 16.5 mM H_3_PO_4_ in 11.5% MeCN, pH 7.1 using NH_4_OH 28%–30%. The gradient used was 0% B over 8.4 min, then 100% B at 8.5 min for 10 min, 0% B for 9 min before the next injection. The injection volume was 10 μL. The running time was 30 min, with a flow rate adjusted at 0.8 mL/min. The column eluate was combined using a tee connector with the oxidant: 100 mM H_3_PO_4_, 5 mM H_5_IO_6_ aqueous solution adjusted to pH 7.8 with 5 M NaOH; the oxidant flow rate was set at 0.5 mL/min. The resulting mix was heated while passing through a knitted reaction coil (teflon tube, 5 m × 0.50 mm i.d.) from Supelco (Madrid, Spain) immersed in a water bath at 80 °C. The eluate from the reaction coil was combined using a tee connector with 0.1 M nitric acid at a flow rate of 0.3 mL/min, to reach a pH outflow ranging between 5–7 [21]. Finally, the fluorescent eluted derivatives were monitored using a fluorescence detector at 330 and 395 nm excitation/emission wavelengths, respectively.

### 3.4. Calculation of the Concentration of GTX4

Identification of PSTs was done comparing retention times between the samples and standards.

GTX1 was quantified directly, the reductive reaction was not needed for quantification. Consequently, the concentration was calculated using the linear regression of the calibration curve for this toxin and the results were corrected for the method dilution [13]. The equation for calculation of final concentration is:

μM_GTX1_ = (μM × Fvol/Ext.vol) × DF) (1)

where μM_GTX1_ = μmol/L, final concentration of GTX1 in the extract; μM = μmol/L, concentration obtained from calibration curve; Fvol = final volume of the deproteinized extract (560 μL); Ext.vol = volume of crude extract used (500 μL); DF is dilution factor = 2, derived from 5 g of tissue extracted with 10 mL.

The concentration of GTX4 was calculated after reductive reaction, from the concentration of NEO formed, which was calculated from the linear regression curve (peak area *versus* concentration in μM) with the results for injections of the standard solutions of NEO.

The concentration of NEO, as determined after GTX toxins conversion, is equal to the sum of GTX1 and GTX4 in a 100% effective reaction. Therefore:

μM_NEO_ = μM for GTX1 + μM for GTX4 (2)

The reduction reaction involves a dilution of the sample, 22 μL of sample to 200 μL of final volume; however, the conversion is not complete, only 82.2% is transformed into NEO. These two factors should be used to correct the concentration of NEO. The concentration must be corrected again with dilutions factors inherent to PCOX method as shown in Equation (1).

μM_NEO_ = (μM × (Fre/Ext.re) × (100/82.2)) × (Fvol/Ext.vol) × DF (3)

where μM_NEO_ = μmol/L, final concentration of NEO in the sample; μM = μmol/L, obtained using the linear regression of the calibration curve; Fre = final volume of the reaction mixture (200 μL); Ext.re = volume of extract used in the reaction (22 μL); 100/82.2 = recovery of reduction reaction; Fvol = final volume of the deproteinized extract (560 μL); Ext.vol = volume of extract used (500 μL); DF is dilution factor = 2, derived from 5 g of tissue extracted with 10 mL.

Combining Equations (2) and (3) obtains a result of:

μM_GTX1_ + μM_GTX4_ = (μM × (Fre/Ext.re) × (100/82.2)) × (Fvol/Ext.vol) × DF (4)

If GTX1 was previously detected in the sample, it was necessary to subtract its concentration to reduce the potential for overestimation of GTX4:

μM_GTX4_ = ((μM × (Fre/Ext.re) × (100/82.2)) × (Fvol/Ext.vol) × DF) − μM_GTX1_ (5)

If GTX1 was not detected in the sample, the concentration obtained in Equation (4) corresponds only to GTX4.

## 4. Conclusions

In summary, each matrix is different and greatly influences the toxins elution pattern. With regard to routine monitoring analyses, these various chromatographic behaviors pose some issues in the identification or quantification of erroneous toxins. In this study, a GTX4 reductive reaction to NEO is being proposed. This new protocol is a less time-consuming and economical alternative to solving the co-eluting peak problem between natural fluorescence matrix interference and toxin peaks.

## References

[1] Alexander J., Auðunsson G., Benford D., Cockburn A., Cravedi J., Dogliotti E., di Domenico A., Férnandez-Cruz M., Fürst P., Fink-Gremmels J. (2008). Cross-contamination of non-target feedingstuffs by monensin authorised for use as a feed additive. EFSA J..

[2] Rourke W.A., Murphy C.J., Pitcher G., van de Riet J.M., Burns B.G., Thomas K.M., Quilliam M.A. (2008). Rapid postcolumn methodology for determination of paralytic shellfish toxins in shellfish tissue. J. AOAC Int..

[3] Rodrigues S.M., de Carvalho M., Mestre T., Ferreira J.J., Coelho M., Peralta R., Vale P. (2012). Paralytic shellfish poisoning due to ingestion of gymnodinium catenatum contaminated cockles—Application of the AOAC HPLC official method. Toxicon.

[4] Meyer K., Sommer H., Schoenholz P. (1928). Shellfish poisoning. Medicine.

[5] Bandem D., Trainer V., Falconer I.R. (1993). Mode of action of toxins of seafood poisoning. Algal Toxins in Seafood and Drinking Water.

[6] Silva M., Barreiro A., Rodriguez P., Otero P., Azevedo J., Alfonso A., Botana L.M., Vasconcelos V. (2013). New invertebrate vectors for pst, spirolides and okadaic acid in the north atlantic. Mar. Drugs.

[7] Anon A. (2005). Official method 959.08. Paralytic shellfish poison. Biological method. Final action. AOAC Off. Methods Anal..

[8] Alexander J., Benford D., Boobis A., Ceccatelli S., Cravedi J.-P., Di A., Domenico D.D., Dogliotti E., Edler L., Farmer P. (2009). Marine biotoxins in shellfish—Summary on regulated marine biotoxins scientific opinion of the panel on contaminants in the food chain. EFSA J..

[9] Anon A. (2005). Official Method 2005.06 Quantitative Determination of Paralytic Shellfish Poisoning Toxins in Shellfish Using Pre-Chromatographic Oxidation and Liquid Chromatography with Fluorescence Detection.

[10] Anon A. (2011). Official Method 2011.02 Determination of Paralytic Shellfish Poisoning Toxins in Mussels, Clams, Oysters and Scallops.

[11] DeGrasse S.L., van de Riet J., Hatfield R., Turner A. (2011). Pre-versus post-column oxidation liquid chromatography fluorescence detection of paralytic shellfish toxins. Toxicon.

[12] Rey V., Alfonso A., Botana L.M., Botana A.M. (2015). Influence of different shellfish matrices on the separation of PSP toxins using a postcolumn oxidation liquid chromatography method. Toxins.

[13] Van de Riet J., Gibbs R.S., Muggah P.M., Rourke W.A., MacNeil J.D., Quilliam M.A. (2010). Liquid chromatography post-column oxidation (PCOX) method for the determination of paralytic shellfish toxins in mussels, clams, oysters, and scallops: Collaborative study. J. AOAC Int..

[14] Li A., Ma J., Cao J., Wang Q., Yu R., Thomas K., Quilliam M. (2012). Analysis of paralytic shellfish toxins and their metabolites in shellfish from the north yellow sea of china. Food Addit. Contam. A.

[15] Baker T.R., Doucette G.J., Powell C.L., Boyer G.L., Plumley F.G. (2003). Gtx(4) imposters: Characterization of fluorescent compounds synthesized by *pseudomonas stutzeri* SF/PS and *pseudomonas*/*alteromonas* PTB-1, symbionts of saxitoxin-producing *alexandrium* spp.. Toxicon.

[16] Sato S., Sakai R., Kodama M. (2000). Identification of thioether intermediates in the reductive transformation of gonyautoxins into saxitoxins by thiols. Bioorg. Med. Chem. Lett..

[17] Sakamoto S., Sato S., Ogata T., Kodama M. (2000). Formation of intermediate conjugates in the reductive transformation of gonyautoxins to saxitoxins by thiol compounds. Fish. Sci..

[18] Oshima Y., Lassus P., Arzul G. (1995). Chemical and enzymatic transformation of paralytic shellfish toxins in marine organisms. Harmful Marine Algal Blooms.

[19] Asakawa M., Takagi M., Iida A., Oishi K. (1987). Studies on the conversion of paralytic shellfish poison (PSP) components by biochemical reducing agents. Jpn. J. Toxicol. Environ. Health.

[20] Shimizu Y., Yoshioka M. (1981). Transformation of paralytic shellfish toxins as demonstrated in scallop homogenates. Science.

[21] Vale C., Alfonso A., Vieytes M.R., Romarís X.M., Arévalo F., Botana A.M., Botana L.M. (2008). *In vitro* and *in vivo* evaluation of paralytic shellfish poisoning toxin potency and the influence of the pH of extraction. Anal. Chem..

